# Enzymatic Glycosylation of *Ganoderma* Terpenoid via Bacterial Glycosyltransferases and Glycoside Hydrolases

**DOI:** 10.3390/biom15050655

**Published:** 2025-05-01

**Authors:** Te-Sheng Chang, Jiumn-Yih Wu, Hsiou-Yu Ding, Tzi-Yuan Wang

**Affiliations:** 1 Department of Biological Sciences and Technology, National University of Tainan, Tainan 70005, Taiwan; mozyme2001@gmail.com; 2 Department of Food Science, National Quemoy University, Kinmen 89250, Taiwan; wujy@nqu.edu.tw; 3 Department of Cosmetic Science, Chia Nan University of Pharmacy and Science, Tainan 717301, Taiwan; ding8896@gmail.com; 4 Biodiversity Research Center, Academia Sinica, Taipei 11529, Taiwan

**Keywords:** biotransformation, enzymatic synthesis, glycosylation, glycosyltransferase, glycoside hydrolase, glucoside, triterpenoids, phenolics, GT1, GH

## Abstract

Glycosylation is a critical enzymatic modification that involves the attachment of sugar moieties to target compounds, considerably influencing their physicochemical and biological characteristics. This review explored the role of two primary enzyme classes—glycosyltransferases (GTs) and glycoside hydrolases (GHs, glycosidases)—in catalyzing the glycosylation of natural products, with a specific focus on *Ganoderma* triterpenoids. While GTs typically use activated sugar donors, such as uridine diphosphate glucose, certain GHs can leverage more economical sugar sources, such as sucrose and starch, through transglycosylation. This paper also reviewed strategies for producing novel terpenoid glycosides, particularly recently isolated bacterial GTs and GHs capable of glycosylating terpenoids and flavonoids. It summarized the newly synthesized glycosides’ structures and biotransformation mechanisms, enhanced aqueous solubility, and potential applications. The regioselectivity and substrate specificity of GTs and GHs in catalyzing *O*-glycosylation (glucosylation) at distinct hydroxyl and carboxyl groups were compared. Furthermore, a special case in which the novel glycosylation reactions were mediated by GHs, including the formation of unique glycoside anomers, was included. The advantages and specific capabilities of GT/GH enzymes were evaluated for their potential in biotechnological applications and future research directions. Novel fungal triterpenoid glycosides produced through various glycosidases and sugars is expected to expand their potential applications in the future.

## 1. Introduction

**Figure 1:**
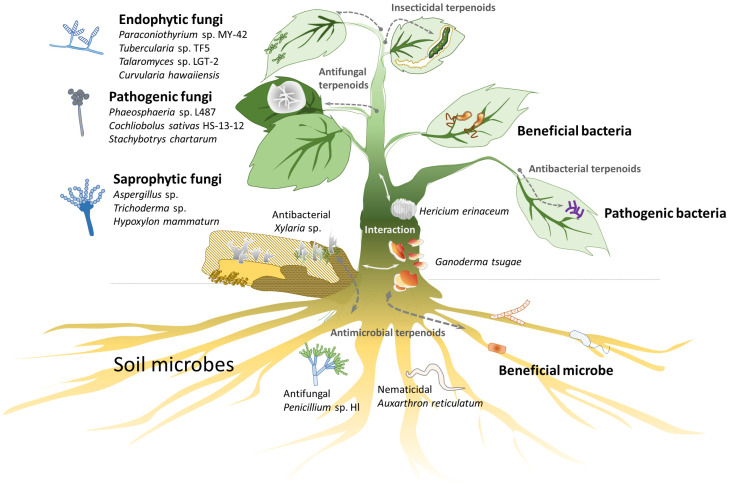
The terpenoid glycosides can be isolated from the natural environment, including plants, soil, and decaying wood. Both terpenoids and terpenoid glycosides form part of the antimicrobial defense systems in plants and fungi. Compared with the large amount of terpenoid glycosides identified from plants or animals [6,7,8,9], the natural terpenoid glycosides from fungi are relative rare. The fungal terpenoid glycosides can be classified into three groups: (1) sesquiterpenoid glycosides (C15) isolated from *Trichoderma asperellum* [10], *Cochliobolus sativus* [11], *Stachybotrys chartarum* [12], and *Aspergillus sydowii* [13]; (2) diterpenoid glycosides (C20) from *Xylotumulus gibbisporus*, *Curvularia hawaiiensis*, and *Penicillium* sp., which exhibit antifungal activity [14,15]; and (3) triterpenoid glycosides (C30) from *Xylaria* sp., which demonstrate antibacterial activity [16,17].

Natural products, such as terpenoids and phenolics, often exhibit significant biological activities, making them valuable for pharmaceutical, cosmeceutical, and nutraceutical applications [1,2]. Terpenoids are particularly abundant in plants and fungi [3,4,5]. Although they do not have a direct relationship with plant growth and development, terpenoids serve as important secondary metabolites of plants, are critical for plant–environment interactions, and provide resistance to cold, water shortages and defense against diseases and pests (Figure 1).

**Figure 2:**
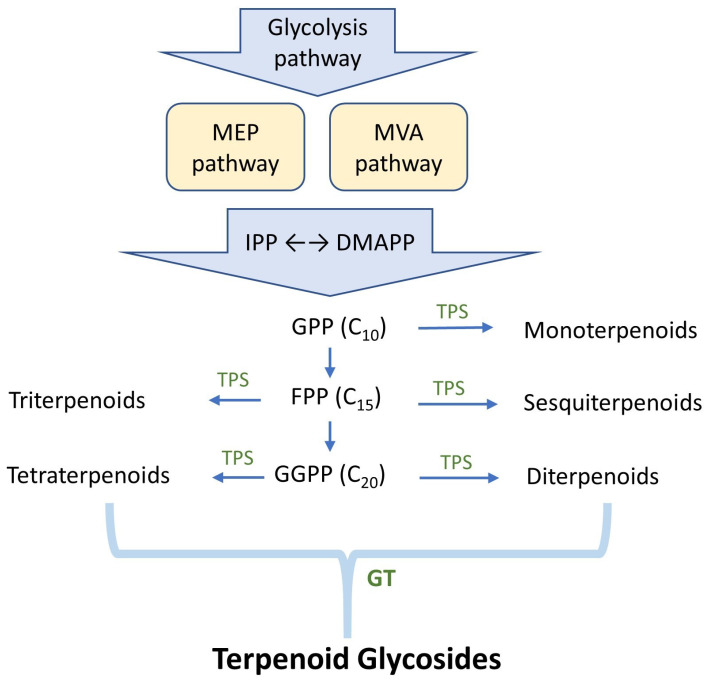
Biosynthetic pathway of terpenoid glycosides. MEP: 2-C-methylerythritol 4-phosphate pathway; MVA: mevalonate pathway; IPP: isopentenyl pyrophosphate; DMAPP: dimethylallyl pyrophosphate; GPP: geranyl pyrophosphate; FPP: farnesyl pyrophosphate; GGPP: geranylgeranyl pyrophosphate; TPS: terpene synthase; GT: glycosyltransferase.

Based on the number of isoprene units, terpenoids are classified into monoterpenoids (C10), sesquiterpenoids (C15), diterpenoids (C20), sesterterpenoids (C25), triterpenoids (C30), and tetraterpenoids (C40). These diverse terpenoids are synthesized from two universal five-carbon precursors: isopentenyl pyrophosphate and its isomer dimethylallyl pyrophosphate (Figure 2) [18]. In nature, these precursors are synthesized via two upstream biosynthetic pathways: the mevalonate and methylerythritol pathways. Both compounds are ligated in downstream pathways to produce intermediate pyrophosphates, such as geranyl pyrophosphate, farnesyl pyrophosphate, and geranylgeranyl pyrophosphate. These pyrophosphates are then modified via numerous terpene synthases to produce various natural terpenoids [19]. After formation, some terpenoids are glycosylated by glycosyltransferases (GTs) to form terpenoid glycosides (Figure 2).

Glycosylation, the process of covalently attaching a carbohydrate to another molecule, is a common strategy in nature to enhance the solubility, stability, and bioactivity of secondary metabolites. Enzymatic glycosylation, using biocatalysts such as GTs and glycoside hydrolases (GHs, glycosidases), offers several advantages over chemical methods, including high regioselectivity, substrate specificity, and mild reaction conditions. Both GTs and GHs have been applied in the glycosylation of natural products, such as phenolics and plant terpenoids, in recent decades [20,21,22,23,24,25,26,27,28,29,30,31,32,33]. This review focuses on recent advances in the enzymatic glycosylation of fungal (*Ganoderma*) triterpenoids using bacterial GTs and GHs to produce novel glycosides with potentially improved properties.

Terpenoid glycosides are abundantly found in plants [7] and many diterpenoid glycosides are also common in animals, such as gorgonian corals [6]. However, terpenoid glycosides are rarely found in fungi [5]. For example, *Ganoderma lucidum* (in Chinese, “Lingzhi”) has been used as a nutritional supplement due to the immunomodulatory and antitumor activities of its polysaccharides and triterpenoids [34]. The medical fungus genus *Ganoderma* contains more than 374 triterpenoids [34], and *Aspergillus* contains more than 288 triterpenoids; however, they have only three triterpenoid glycosides each [35]. This disparity indicates that fungi may lack specific GTs to form terpenoid glycosides [36].

**Figure 3:**
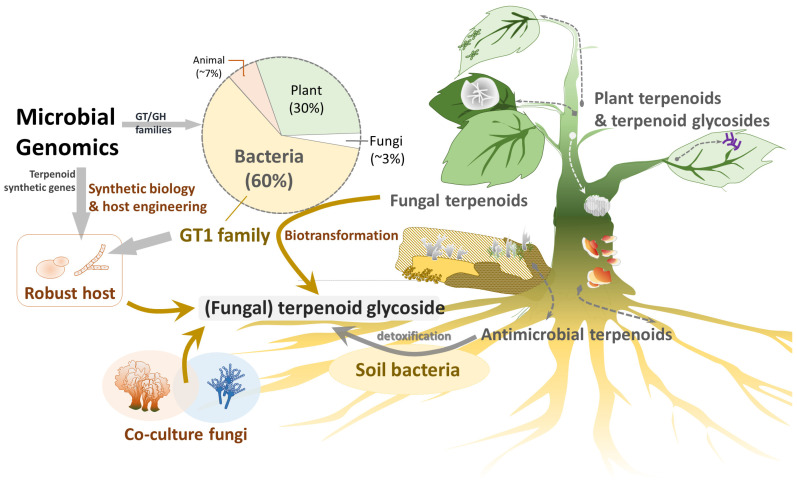
Strategies for searching for novel terpenoid glycosides. Bacterial GTs may biotransform plant and fungal metabolites, such as terpenoids and phenolics, into glycosides, which may alter the activity of the original metabolites in terms of solubility, bioavailability, and detoxification. In practice, these enzymes can be assessed by combining novel bacterial GT and GH genes with gene clusters for aglycone scaffolds in suitable hosts. In addition, co-culturing microbes with natural compounds or precursors may also help isolate novel derivatives.

Fungi and bacteria coexist with plants in ecosystems (Figure 3). To compete for limited carbon resources (sugars), microbes need to obtain sugars quickly and/or kill other microbes. Plants and fungi produce terpenoids as chemical defenses to kill other microbes or insects [37]. Autotrophic plants have diverse terpenoids and terpenoid glycosides, whereas heterotrophic fungi have some terpenoids and few terpenoid glycosides, likely due to the lack of specific GTs.

The GT1 family of enzymes plays a key role in terpenoid glycoside production. According to the Carbohydrate-Active Enzymes (CAZy) database, over 20,000 GT1 have been identified [36], with nearly 60% originating from bacteria.

The remaining 30% and approximately 7% originate from plants and animals, respectively [36]. In contrast, only a few GT1 genes originate from fungi, likely reflecting the paucity of terpenoid glycosides in fungi. On the other hand, GT1 family genes are quite commonly found in bacteria. This may be because the bacteria need the GT1 enzyme to detoxify the antibacterial terpenoids into terpenoid glycosides for survival. Notably, macrolide-producing *Streptomyces lividans* glycosylates antibiotics via GT1 enzymes for self-protection [38]. Recent studies have demonstrated that GT1 enzymes confer resistance to toxic terpenoids by glycosylating them [39,40,41].

There may be two reasons why the heterotrophic fungi lose GT1 family genes and mainly produce terpenoids. First, fungi, competing with plants and microbes for limited sugar (carbon) sources, may have evolutionarily lost terpenoid glycosylation pathways or related metabolisms to conserve energy. This hypothesis is supported by the rarity of glycosylated secondary metabolites, including phenols, terpenes, alkaloids, and terpenoids, in fungi [18,34,35,42,43,44,45,46,47,48,49,50]. Second, terpenoid glycosides might be toxic to fungi. Thus, fungi do not waste energy synthesizing these types of toxic compounds. Some terpenoid glycosides possess amphiphilic properties, and the membrane-permeabilizing property induces cell toxicity in advance [7]. Some plant- and animal-derived terpenoid glycosides exhibit antifungal activity, providing protection from pathogenic fungi. Notably, a few fungal terpenoid glycosides have been reported to exhibit weak antibacterial activity [10].

## 2. Strategies for Producing Novel Terpenoid Glycosides

One approach to increasing terpenoid glycoside production is to cultivate heterotrophic fungi enriched with sugars or to integrate bacterial GT genes into the genomes of terpenoid-producing fungi. The recombinant fungi could then glycosylate cellular terpenoids to produce terpenoid glycosides with functionally expressed GTs. However, if these terpenoid glycosides prove to be toxic to the fungal host, this strategy may not be viable. Alternatively, screening to identify a robust host and integrating related pathway genes are required to mitigate toxicity and produce terpenoid glycosides.

Synthetic biology, encompassing techniques such as metabolic engineering and genome editing, offers solutions for the sustainable and efficient production of high-value plant terpenoids in industrial microbial cell factories, *Escherichia coli* and *Saccharomyces cerevisiae* [51,52,53]. These genetic tools have been applied to produce various pharmaceutical terpenoids (terpenoid glycosides) on a large scale. However, the biosynthetic pathway genes of fungal terpenoids remain insufficiently understood.

Many studies have described the entire biosynthetic pathways of plant terpenoids or terpenoid glycosides, but knowledge of fungal biosynthetic pathways remains limited. Comparative genomics between closely related fungi with and without terpenoid glycosides may help identify putative biosynthetic pathway genes of fungal terpenoids (terpenoid glycosides), such as GTs responsible for terpenoid glycoside synthesis. For example, Basidiomycete *Hericium erinaceus* produces diverse erinacine diterpenoid glycosides [54]. Recently, a terpenoid biosynthetic gene cluster was identified in *H. erinaceus*, comprising ten synthetic pathway genes (*eriA* to *eriJ*), including one GT (*eriJ*) [54]. By using a novel knock-in genetic tool to integrate these genes into Ascomycete *Aspergillus oryzae*, recombinant *A. oryzae* successfully produced erinacine Q diterpenoid glycosides in vivo [55].

Furthermore, only small portions of the secondary metabolites have been identified in pure-cultured fungi, suggesting that co-culture systems may be better suited to produce more secondary metabolites, such as fungal terpenoid glycosides, because microbes may compete with limited resources to survive in natural environments. Such interspecific interactions may activate the biosynthesis of unique secondary metabolites. For example, *Xylaria flabelliformis* produced an extra secondary metabolite, wheldone, only when co-cultured with *Aspergillus fischeri* [56]. Similarly, *Micromonospora* species also produced an extra antibiotic, keyicin, only when co-cultured with a *Rhodococcus* species [57]. Therefore, a co-culturing system is another approach to isolating more terpenoid glycosides.

## 3. Bacterial GT/GH Enzymes for New *Ganoderma* Terpenoid Glycosides

Because nearly 60% of GT1 genes originate from bacteria, recombinant bacterial GT1 enzymes could be applied to biotransform valuable but low-soluble terpenoids. A notable example is *Ganoderma lucidum* (Reishi mushroom), which has long been used in traditional medicine due to its diverse pharmacological activities [34]. These activities are largely attributed to its triterpenoid constituents, particularly ganoderic acids (GAs), which exhibit various bioactivities, including anti-inflammatory, antitumor, and antioxidant effects. However, the poor aqueous solubility, and thus bioavailability, of some GAs limits their clinical application.

Glycosylation is a strategy to overcome these limitations [58]. There are two glycosylation methods: chemical synthesis and enzymatic biotransformation. The main difficulty with the chemical synthesis of terpenoid glycosides is the lack of regioselectivity of multiple hydroxyl groups in the sugar moiety. In contrast, biotransformation is a promising method because of the regioselective and enantioselective synthesis of bioactive compounds [25,30,32,59,60,61,62].

**Figure 4:**
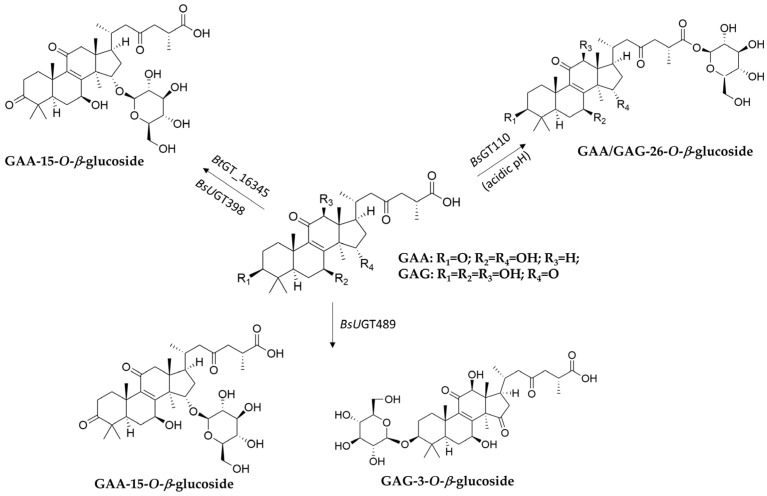
Glycosylation of ganoderic acid A (GAA) and ganoderic acid G (GAG) by *Bacillus* GTs.

**Figure 5:**
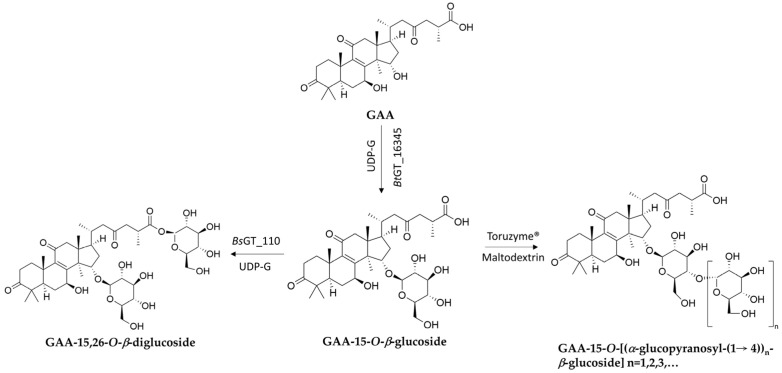
One-pot bienzymatic cascade synthesis via the combination of *Bt*GT_16345 and other enzymes to produce GAA-disaccharides. UDP-G: uridine diphosphate glucoside.

**Figure 6:**
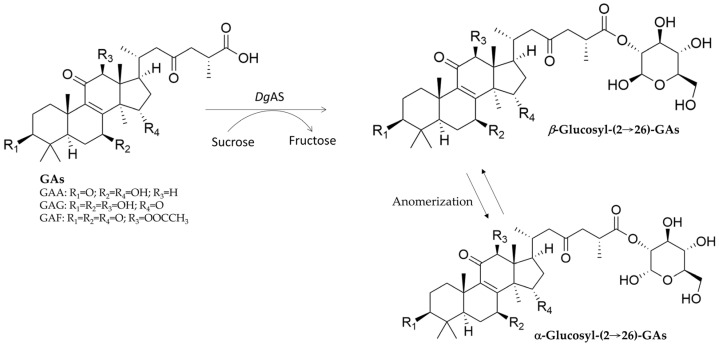
Biotransformation of three ganoderic acids (GAs) into glucose-linked GAs by *Dg*AS [74].

Although GT- or GH-mediated glycosylation of natural products (mainly in phenolics) has been reported [20,21,22,23,24,25,26,27,28,29,30,31,32,33], few terpenoid glycosides have been biotransformed from terpenoids. Therefore, this section highlights GTs or GHs that can generate new terpenoid glycosides from fungal terpenoids through enzymatic biotransformation (Table 1) and reviews their glycosylation mechanisms (Figure 4, Figure 5 and Figure 6). These enzymatic modifications can enhance solubility, stability, and bioavailability, and even alter bioactivity [58,63,64,65,66].

**Table 1:** Enzymatic synthesis of new *Ganoderma* terpenoid glycosides.

Enzyme Type	Enzyme	Precursor	Product	Property of the New Glycosides	Illustration	References
Glycosyltransferase (GT)	*Bs*UGT489 ^1,2^, *Bs*UGT398 ^1,2^, *Bt*BT_16345 ^1,3^	Ganoderic acid A (GAA)	GAA-15-*O*-*β*-glucoside	Improved solubility	Figure 4	[67,68,69]
	*Bs*GT110 ^1,2^	GAA	GAA-26-*O*-*β*-glucoside	Improved solubility	Figure 4	[70]
	*Bs*UGT489	Ganoderic acid G (GAG)	GAG-3-*O*-*β*-glucoside	Improved solubility	Figure 4	[71]
	*Bs*GT110	GAG	GAG-26-*O*-*β*-glucoside	Improved solubility	Figure 4	[71]
	Combination of *Bt*BT_16345 and *Bs*GT110	GAA	GAA-15,26-*O*-*β*-diglucoside	Improved solubility	Figure 5	[72]
Glycoside hydrolase (GH)	Combination of *Bt*BT_16345 and Toruzyme ^4^	GAA	GAA-15-*O*-[*α*-glucopyranosyl-(1→4)-*β*-glucopyranoside]	Improved solubility	Figure 5	[73]
	*Dg*AS ^1,5^	GAA	Glucosyl-(2→26)-GAA anomers	Unique anomers	Figure 6	[74]
	*Dg*AS	GAG	Glucosyl-(2→26)-GAG anomers	Unique anomers	Figure 6	[74]
	*Dg*AS	Ganoderic acid F (GAF)	Glucosyl-(2→26)-GAF anomers	Unique anomers	Figure 6	[74]

^1^ Recombinant enzyme isolated from *Escherichia coli*. ^2^ Genetic source: *Bacillus subtilis* ATCC 6633. ^3^ Genetic source: *Bacillus thuregenesis* GA A07. ^4^ Source: *Thermoanaerobacter* species. ^5^ Genetic source: *Deinococcus geothermalis.*

### 3.1. New Terpenoid Glycosides from GT-Catalyzed Biotransformation

GTs (EC 2.4.x.y) catalyze the transfer of sugar moieties from activated donor molecules, typically uridine diphosphate (UDP) sugars such as UDP glucose (UDP-G), to specific acceptor molecules. The CAZy database classifies GTs into numerous families [75]. Among these, the GT1 family includes many enzymes that use small molecules, such as flavonoids and terpenes, as sugar acceptors [36].

Enzymatic biotransformation is a synthetic method with good bioconversion efficiency. However, the difficulty is finding functional enzymes. One approach for identifying suitable enzymes is to feed the microbes with triterpenoids, check whether they can catalyze the precursors, and then screen for the putative enzymes involved in the biotransformation. For example, *Bacillus subtilis* ATCC 6633 was found to glycosylate ganoderic acid A (GAA), a lanostane triterpenoid from *G. lucidum*, and to produce two GAA derivatives: GAA-15-*O*-*β*-glucoside [68] and GAA-26-*O*-*β*-glucoside [70]. Based on the CAZy database, the GTs, which usually belong to GT family 1 (GT1), are responsible for GAA glycosylation. Thus, nine suitable GT candidates were selected from the whole genome sequence of *B. subtilis* ATCC 6633. These candidates were further cloned and overexpressed in *Escherichia coli* to produce pure GT enzymes. After functional screening, three GTs—*Bs*GT110, *Bs*UGT398, and *Bs*UGT489—were confirmed to have regioselective glycosylation activity toward GAA (Figure 4). In addition, *Bs*GT110 was found to catalyze a novel acidic glycosylation of GAA at the C-26 carboxyl group, producing GAA-26-*O*-*β*-glucoside. *Bs*GT110 exhibited optimal activity at pH 6.0 and lost most of its activity at pH 8.0. Kinetic analysis indicated that the catalytic activity of *Bs*GT110 toward GAA was higher at acidic pH. *Bs*GT110 was also shown to glycosylate ganoderic acid G (GAG) at the C-26 position. These findings establish *Bs*GT110 as the first identified GT, catalyzing triterpenoid glycosylation at the C-26 carboxyl group under acidic conditions. Moreover, *Bs*GT110 and *Bs*UGT489 were also found to glycosylate GAG into GAG-26-*O*-*β*-glucoside and GAG-3-*O*-*β*-glucoside, respectively [71].

Similarly, an intestinal *Bacillus thuringiensis* strain, GA A07, from zebrafish exhibited 10-fold higher efficiency for producing GAA-15-*O*-*β*-glucoside than *B. subtilis* ATCC 6633 [67]. A genome-centric approach and functional validation revealed a key glycosyltransferase, *Bt*GT_16345, as the first GT family 28 (GT28) enzyme responsible for regioselective glycosylation on the C-15 hydroxyl group of GAA (Figure 5). The optimal conditions for *Bt*GT_16345 activity were determined to be pH 7.5 and 30 °C, with magnesium ions. Unlike *Bs*UGT398 and *Bs*UGT489, *Bt*GT_16345 showed broader substrate specificity, exhibiting glycosylation activity toward several flavonoids and antcin K [67]. Kinetic studies revealed comparable catalytic efficiencies between *Bt*GT_16345 and *Bs*UGT398/*Bs*UGT489 for GAA glycosylation. Moreover, the highly efficient and highly regioselective glycosylation of *Bt*GT_16345 toward GAA can be integrated with other GTs to form in vitro cascade reactions and produce soluble terpenoid oligosaccharides (Figure 5) [72]. These terpenoid disaccharides possessed several thousand-fold higher solubilities than their parent terpenoid aglycone, implying promising medical applications.

### 3.2. New Terpenoid Glycosides from GH-Catalyzed Biotransformation

In nature, terpenoid glycosides are mainly produced by GT catalytic reactions in the presence of a sugar donor, such as UDP-G [25,60]. However, the high cost of UDP-G limits large-scale industrial applications. Thus, researchers have tried to find alternative enzymes to glycosylate terpenoid aglycones. Several GH enzymes have been identified to glycosylate flavonoids by using cheaper sugar donors, such as starch, cyclodextrin, sucrose, and maltose [32,76]. Although such an affordable biotransformation would be highly useful and scalable, most identified GHs cannot directly glycosylate terpenoid aglycones into terpenoid glycosides [74]. To date, only one GH—*Deinococcus geothermalis* amylosucrase (*Dg*AS)—has been reported to glycosylate fungal terpenoid aglycones. *Dg*AS has been found to glycosylate three triterpenoids from *G. lucidum*, namely, GAA, GAG, and ganoderic acid F (GAF), forming glucosyl-(2→26)-GA anomers with sucrose as the sugar donor (Figure 6) [74]. This activity was optimal under acidic conditions (pH 5–6) and was significantly reduced at neutral or alkaline pH and produces *α*-glucosyl-(2→26)-GAF and *β*-glucosyl-(2→26)-GAF anomers in a 3:2 ratio, as confirmed with nuclear magnetic resonance analysis. Similar anomers were also produced with GAA and GAG.

Natural glycosides are linked by glycosidic bonds, in which the linkages connect the C-1 bounded anomeric carbon of the glucose donor with the sugar acceptor molecules; therefore, either *α*- or *β*-glycosides are formed. However, *Dg*AS causes glycosylation at the C-2 position, with C-1 remaining free and thus anomerizing in solutions, producing both *α*- and *β*-glucose linked GAs. Although unknown, the proposed mechanism involves a carbon switch, leading to a novel glucose linkage. The bioactivity and biological implications of these glucose GAs anomers remain unknown.

Another GH13 enzyme, cyclodextrin glucanotransferase (CGTase, Toruzyme 3.0 L), was used in a sequential biotransformation strategy (Figure 5) [73]. In a one-pot reaction, GAA was first glycosylated at the C-15 position by *Bt*GT_16345 to produce GAA-15-*O*-*β*-glucopyranoside (GAA-15-G). Subsequently, Toruzyme catalyzed the glycosylation of GAA-15-G using maltose, yielding several GAA glucosides: GAA-G2, GAA-G3, and GAA-G4. The major product, GAA-15-*O*-[α-glucopyranosyl-(1→4)-*β*-glucopyranoside] (GAA-G2), exhibited a significantly higher aqueous solubility (>4500-fold) than GAA. Toruzyme showed specificity toward GAA-15-G, as it did not directly glycosylate GAA. The enzyme catalyzed the α-(1→4) glycosylation linkage, which is consistent with previous studies on flavonoid and steroidal saponin glycosylation [77,78,79]. This demonstrated the potential of combining GTs and GHs to synthesize triterpenoid saponins with improved properties.

## 4. Bacterial GTs/GHs Applied to Other Natural Compound Derivatives

**Figure 7:**
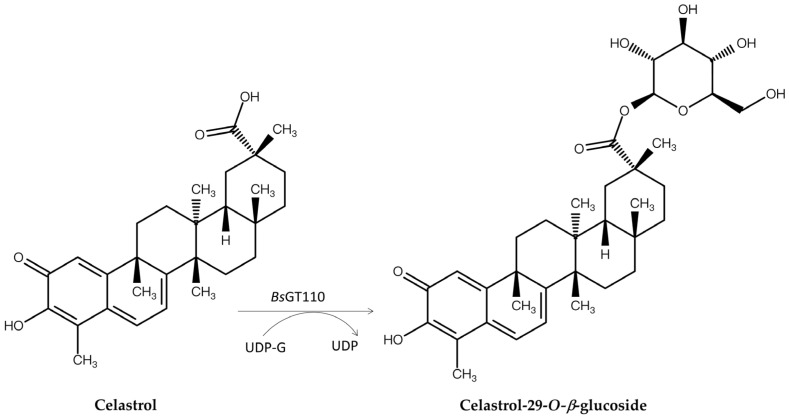
Glycosylation of celastrol to celastrol-29-*O*-*β*-glucoside by *Bs*GT110.

Glycosylation improves solubility [64,66,72,80,81], bioavailability [58], and detoxification [82,83] of molecules, making it a valuable strategy for modifying natural products. Over the past few decades, GTs and GHs have been widely applied in glycosylating various natural products, including phenolic compounds [20,21,22,23,24,25,26,27,28,29,30,31,32,33]. As mentioned, bacterial GTs and GHs can glycosylate *Ganoderma* triterpenoids to produce new terpenoid glycosides with better solubility but lower bioactivity. These bacterial enzymes might also reduce the toxicity of terpenoids. To explore this potential, these recombinant enzymes were applied to celastrol (Figure 7), a pentacyclic triterpenoid isolated from the root of the plant *Tripterygium wilfordii* that not only has high toxicity, resulting in a narrow therapeutic window, but also possesses many useful bioactivities, including antioxidant, antibacterial, anticancer, antiobesity, antidiabetic, and anti-inflammatory effects [63]. Moreover, its low water solubility restricts its oral bioavailability, further hindering its clinical application. *Bs*GT110 considerably mitigated these limitations by glycosylating celastrol to the highly soluble celastrol-29-*O*-*β*-glucoside at pH 8, which also displayed lower toxicity than celastrol (Figure 7). This example underscores the advantages, and thus the increased pharmaceutical applicability, of bacterial enzymatic glycosylation (Table 1 and Table 2) in improving water solubility and reducing the toxicity of compounds such as celastrol [84,85].

**Figure 8:**
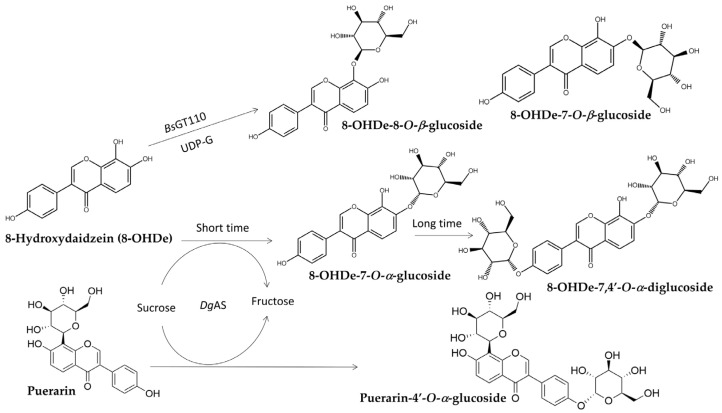
Glycosylation of 8-hydroxydaidzein (8-OHDe) and puerarin by *Bs*GT110 (8-OHDe) or *Dg*AS (8-OHDe, puerarin).

In addition to triterpenoid glycosylation, *Bs*GT110 also catalyzed the glycosylation of soybean isoflavone 8-hydroxydaidzein (8-OHDe) (Figure 8) [86] into two new isoflavone glucosides, 8-OHDe-7-*O*-*β*-glucoside and 8-OHDe-8-*O*-*β*-glucoside, which have significantly higher aqueous solubility (9.0-fold and 4.9-fold, respectively) and improved stability than 8-OHDe. Notably, *Bs*GT110 demonstrated acidic glycosylation activity toward *Ganoderma* triterpenoid GAA at pH 6 [70] but showed optimal glycosylation toward celastrol at pH 8 and isoflavonoid 8-OHDe at pH 7. Thus, the same enzyme showed different optimal reaction conditions toward different substrates, suggesting the importance of identifying optimal reaction conditions for different substrates in future studies on enzymatic synthesis.

*Dg*AS, a GH, glycosylates 8-OHDe into *α*-glucosides, 8-OHDe-7-*O*-*α*-glucopyranoside (8-OHDe-7-G), with a short reaction time [65]. However, prolonged reaction time led to the formation of two additional diglucoside derivatives, indicating *Dg*AS’s broad regioselectivity in forming glycosidic linkages (Figure 8) [88]. The resulting glucosides also exhibited enhanced solubility and stability.

*Dg*AS was also employed for the glycosylation of puerarin, an isoflavone with limited solubility and similar structure to 8-OHDe (Figure 8) [89]. Unlike other GTs and GHs, *Dg*AS glycosylated the 4′-hydroxyl group of puerarin, producing a novel glycoside with improved solubility. This regioselectivity distinguishes it from previously studied GHs, which typically glycosylate the C-glucoside residue of puerarin.

**Figure 9:**
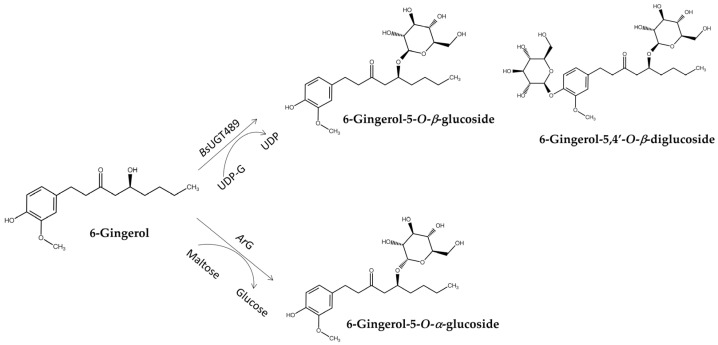
Glycosylation of 6-gingerol to either *β*-glycosides by GT (*Bs*UGT489) or *α*-glycoside by GH (*Ar*G).

*Bs*UGT489 was identified as a suitable GT for the biotransformation of 6-gingerol through glycosylation into several products, namely 6-gingerol-4′,5-*O-β*-diglucoside, 6-gingerol-4′-*O*-*β*-glucoside, and 6-gingerol-5-*O-β*-glucoside (Figure 9) [87]. These glucosides greatly improved aqueous solubility compared with 6-gingerol. In addition, the spontaneous deglucosylation of some 6-gingerol glucosides led to the formation of 6-shogaol derivatives, thereby highlighting the complex interplay between glycosylation and stability. Compared with GTs, another GH, an *α*-glucosidase from *Agrobacterium radiobacter* DSM 30147 (*Ar*G), was found to catalyze the *α*-glycosylation of 6-gingerol to produce 6-gingerol-5-*O*-*α*-glucoside, which possessed 10-fold higher anti-inflammatory activity than 6-gingerol (Figure 9) [80].

**Figure 10:**
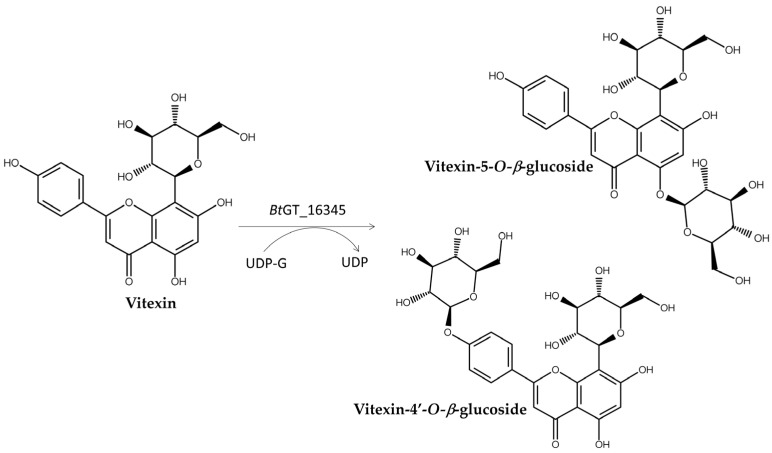
Glycosylation of vitexin to *Bt*GT_16345.

The newly identified GT28 enzyme, *Bs*GT_16345, catalyzed the glycosylation of vitexin, a C-glucoside flavone with low solubility, to produce vitexin-4′-*O*-*β*-glucoside and vitexin-5-*O*-*β*-glucoside, both of which displayed greater aqueous solubility than vitexin (Figure 10) [81].

## 5. Conclusions

The enzymatic glycosylation of natural products using bacterial GTs and GHs has emerged as a powerful strategy for improving their physicochemical properties, particularly aqueous solubility and stability, and potentially enhancing their bioactivity. GTs offer high regioselectivity and typically produce *β*-glucosides using activated sugar donors, such as UDP-G. In contrast, some GHs, such as amylosucrase and maltogenic amylase, can use more cost-effective sugar donors, such as sucrose and starch, to catalyze transglycosylation reactions, facilitating the production of *α*-glucosides. The difficulty of enzymatic glycosylation of fungal triterpenoids has resulted in only a few successful studies of novel *Ganoderma* triterpenoid glycosides being reported. As more scientists focus on this area and more novel fungal triterpenoid glycosides are produced using different glycosidases and sugars, a wider range of applications is expected to emerge in the future.

The enzymatic glycosylation of *Ganoderma* triterpenoids has proven to be promising for synthesizing novel bioactive compounds with improved characteristics. Bacterial GTs, particularly from *Bacillus* species, exhibit high regioselectivity, catalyzing the *O*-glycosylation of ganoderic acids at specific hydroxyl (C-15) and carboxyl (C-26) groups using UDP glucose as a sugar donor. The identification of enzymes such as *Bs*UGT398, *Bs*UGT489, *Bt*GT_16345, and *Bs*GT110 has significantly advanced the modification of these valuable natural products. Furthermore, the application of GHs, such as *Dg*AS and Toruzyme, introduces novel possibilities. *Dg*AS demonstrates a unique ability to form both *α*- and *β*-anomers with a non-anomeric carbon linkage to the C-26 carboxyl group, expanding the structural diversity of *Ganoderma* triterpenoid glycosides. The sequential use of GTs and GHs, as demonstrated by *Bt*GT_16345 and Toruzyme, presents a powerful strategy for producing triterpenoid saponins with significantly enhanced aqueous solubility.

Beyond *Ganoderma* triterpenoids, the glycosylation of phenolic compounds, including 8-OHDe, vitexin, puerarin, mangiferin, corylin, and gingerols, demonstrates the versatility of GTs and GHs in modifying numerous natural compounds. The resulting glycosides often exhibit significantly enhanced aqueous solubility, which is a critical factor in their bioavailability and industry application.

Investigating the bioactivity of fungal terpenoid glycosides is a developing field. An exploration of microbial genomes could uncover new GTs or GHs and terpenoid biosynthetic pathway genes that could be applied to produce non-natural terpenoid glycosides. Additionally, terpenoid biosynthetic pathway genes could be engineered into microorganisms, such as *Ganoderma* or new hosts, to produce additional terpenoid glycosides. Moreover, fungal co-culture systems might also stimulate the discovery of more natural terpenoid glycosides.

Future studies should identify and engineer novel GTs and GHs with improved catalytic efficiency, substrate specificity, and tolerance to reaction conditions. Exploring the regioselectivity of these enzymes and developing methods for controlling the degree of glycosylation are also key areas of investigation. Furthermore, in-depth studies on the bioactivity and bioavailability of newly synthesized glucosides are crucial to fully realizing their potential applications in pharmaceuticals, cosmeceuticals, and other fields. The application of computational tools and predictive methods can aid in the selection of suitable enzyme–substrate pairs and the design of novel glycosylated natural products with desired properties. The continued exploration of enzymatic glycosylation promises to unlock the full potential of natural products for various biomedical and industrial applications.

## Figures and Tables

**Table 2:** Enzymatic glycosylation of plant precursors by glycosyltransferases (GTs) and glycoside hydrolases (GHs).

Enzyme Type	Enzyme Name	Precursor	Product	Property of the New Glycosides	Illustration	References
GT	*Bs*GT110 ^1,2^	Celastrol	Celastrol-29-*O*-*β*-glucoside	Detoxification	Figure 7	[63]
	*Bs*GT110	8-Hydroxydaidzein (8-OHDe)	8-OHDe-7-*O*-*β*-glucoside 8-OHDe-8-*O*-*β*-glucoside	Improved solubility and stability	Figure 8	[86]
	*Bs*UGT489 ^1,2^	6-Gingerol	6-Gingerol-5-*O*-*β*-glucoside 6-Gingerol-5,4′-*O*-*β*-diglucoside	Improved solubility and anti-inflammatory activity	Figure 9	[87]
	*Bt*BT_16345 ^1,3^	Vitexin	Vitexin-5-*O*-*β*-glucoside Vitexin-4′-*O*-*β*-glucoside	Improved solubility	Figure 10	[81]
GH	*Dg*AS ^1,4^	8-OHDe	8-OHDe-7-*O*-*α*-glucoside 8-OHDe-7,4′-*O*-*α*-diglucoside	Improved solubility and stability	Figure 8	[65,88]
	*Dg*AS	Puerarin	Puerarin-4′-*O*-*α*-glucoside	Improved solubility	Figure 8	[89]
	*Ar*G ^1,5^	6-Gingerol	6-Gingerol-5-*O*-*α*-glucoside	Improved stability and anti-inflammatory activity	Figure 9	[80]

^1^ Recombinant enzyme isolated from *Escherichia coli*. ^2^ Genetic source: *Bacillus subtilis* ATCC 6633. ^3^ Genetic source: *Bacillus thuregenesis* GA A07. ^4^ Genetic source: *Deinococcus geothermalis*. ^5^ Genetic source: *Agrobacterium radiobacter.*

## Data Availability

The data presented in this study are available on request from the corresponding author.

## References

[1] Badshah S.L., Faisal S., Muhammad A., Poulson B.G., Emwas A.H., Jaremko M. (2021). Antiviral activities of flavonoids. Biomed. Pharmacother..

[2] Ekiert H.M., Szopa A. (2020). Biological activities of natural products. Molecules.

[3] Couillaud J., Leydet L., Duquesne K., Iacazio G. (2021). The terpene mini-path, a new promising alternative for terpenoids bio-production. Genes.

[4] Boncan D.A.T., Tsang S.S.K., Li C., Lee I.H.T., Lam H.M., Chan T.F., Hui J.H.L. (2020). Terpenes and terpenoids in plants: Interactions with environment and insects. Int. J. Mol. Sci..

[5] de Souza J.J., Vieira I.J., Rodrigues-Filho E., Braz-Filho R. (2011). Terpenoids from endophytic fungi. Molecules.

[6] Berrue F., McCulloch M.W., Kerr R.G. (2011). Marine diterpene glycosides. Bioorganic Med. Chem..

[7] Khan H., Khan Z., Amin S., Mabkhot Y.N., Mubarak M.S., Hadda T.B., Maione F. (2017). Plant bioactive molecules bearing glycosides as lead compounds for the treatment of fungal infection: A review. Biomed. Pharmacother..

[8] Mondol M.A.M., Shin H.J., Rahman M.A., Islam M.T. (2017). Sea cucumber glycosides: Chemical structures, producing species and important biological properties. Mar. Drugs.

[9] Xiao G., Shao X., Zhu D., Yu B. (2019). Chemical synthesis of marine saponins. Nat. Prod. Rep..

[10] Song Y.P., Miao F.P., Liu X.H., Yin X.L., Ji N.Y. (2019). Seven chromanoid norbisabolane derivatives from the marine-alga-endophytic fungus *Trichoderma asperellum* A-YMD-9-2. Fitoterapia.

[11] Li Y.-Y., Tan X.-M., Wang Y.-D., Yang J., Zhang Y.-G., Sun B.-D., Gong T., Guo L.-P., Ding G. (2020). Bioactive seco-Sativene Sesquiterpenoids from an *Artemisia desertorum* Endophytic Fungus, *Cochliobolus sativus*. J. Nat. Prod..

[12] Li Y., Liu D., Cheng Z., Proksch P., Lin W. (2017). Cytotoxic trichothecene-type sesquiterpenes from the sponge-derived fungus *Stachybotrys* chartarum with tyrosine kinase inhibition. RSC Adv..

[13] Liu S., Haibo W., Mingzhi S., Ja H.G., Jongki H., and Jung J.H. (2017). New metabolites from the sponge-derived fungus *Aspergillus sydowii* J05B-7F-4. Nat. Prod. Res..

[14] Zhang M.-Q., Xu K.-X., Xue Y., Cao F., Yang L.-J., Hou X.-M., Wang C.-Y., Shao C.-L. (2019). Sordarin diterpene glycosides with an unusual 1,3-dioxolan-4-one ring from the zoanthid-derived fungus *Curvularia hawaiiensis* TA26-15. J. Nat. Prod..

[15] Wang C., Gao Y.K., Lei F.H., Tan X.C., Shen L.Q., Gao C.H., Yi X.X., Li X.Y. (2019). A new glycosyl ester isolated from marine-derived *Penicillium* sp.. Herbs.

[16] Tang G.-H., Na L., Wei L., Min W., Yun-Yun C., Hai-Ying Z., and He S.-Y. (2020). Mannosylxylarinolide, a new 3,4-seco-ergostane-type steroidal saponin featuring a β-d-mannose from the endophytic fungus *Xylaria* sp.. J. Asian Nat. Prod. Res..

[17] Deyrup S.T., Gloer J.B., O’Donnell K., Wicklow D.T. (2007). Kolokosides A−D:  Triterpenoid glycosides from a Hawaiian isolate of *Xylaria* sp.. J. Nat. Prod..

[18] Wang Q., Cao R., Zhang Y., Qi P., Wang L., Fang S. (2021). Biosynthesis and regulation of terpenoids from basidiomycetes: Exploration of new research. AMB Express.

[19] Chen F., Tholl D., Bohlmann J., Pichersky E. (2011). The family of terpene synthases in plants: A mid-size family of genes for specialized metabolism that is highly diversified throughout the kingdom. Plant J..

[20] Zhao J.N., Wang R.F., Zhao S.J., Wang Z.T. (2020). Advance in glycosyltransferases, the important bioparts for production of diversified ginsenosides. Chin. J. Nat. Med..

[21] Feng Y., Yao M., Wang Y., Ding M., Zha J., Xiao W., Yuan Y. (2020). Advances in engineering UDP-sugar supply for recombinant biosynthesis of glycosides in microbes. Biotechnol. Adv..

[22] Kim B.G., Yang S.M., Kim S.Y., Cha M.N., Ahn J.H. (2015). Biosynthesis and production of glycosylated flavonoids in *Escherichia coli*: Current state and perspectives. Appl. Microbiol. Biotechnol..

[23] Ren J., Barton C.D., Zhan J. (2023). Engineered production of bioactive polyphenolic *O*-glycosides. Biotechnol. Adv..

[24] Overwin H., Wray V., Seeger M., Sepulveda-Boza S., Hofer B. (2016). Flavanone and isoflavone glucosylation by non-Leloir glycosyltransferases. J. Biotechnol..

[25] Mrudulakumari Vasudevan U., Lee E.Y. (2020). Flavonoids, terpenoids, and polyketide antibiotics: Role of glycosylation and biocatalytic tactics in engineering glycosylation. Biotechnol. Adv..

[26] Moulis C., Andre I., Remaud-Simeon M. (2016). GH13 amylosucrases and GH70 branching sucrases, atypical enzymes in their respective families. Cell. Mol. Life Sci..

[27] Nidetzky B., Gutmann A., Zhong C. (2018). Leloir glycosyltransferases as biocatalysts for chemical production. ACS Catal..

[28] Mestrom L., Przypis M., Kowalczykiewicz D., Pollender A., Kumpf A., Marsden S.R., Bento I., Jarzebski A.B., Szymanska K., Chrusciel A. (2019). Leloir glycosyltransferases in applied biocatalysis: A multidisciplinary approach. Int. J. Mol. Sci..

[29] Tian Y., Xu W., Zhang W., Zhang T., Guang C., Mu W. (2018). Amylosucrase as a transglucosylation tool: From molecular features to bioengineering applications. Biotechnol. Adv..

[30] Herrera-Gonzalez A., Nunez-Lopez G., Morel S., Amaya-Delgado L., Sandoval G., Gschaedler A., Remaud-Simeon M., Arrizon J. (2017). Functionalization of natural compounds by enzymatic fructosylation. Appl. Microbiol. Biotechnol..

[31] Moulis C., Guieysse D., Morel S., Severac E., Remaud-Simeon M. (2021). Natural and engineered transglycosylases: Green tools for the enzyme-based synthesis of glycoproducts. Curr. Opin. Chem. Biol..

[32] Slamova K., Kapesova J., Valentova K. (2018). “Sweet flavonoids”: Glycosidase-catalyzed modifications. Int. J. Mol. Sci..

[33] Zhang L., Xu Q., Zhu J., Xia G., Zang H. (2021). Synthesis, alpha-glucosidase inhibition and molecular docking studies of tyrosol derivatives. Nat. Prod. Res..

[34] Xia Q., Zhang H., Sun X., Zhao H., Wu L., Zhu D., Yang G., Shao Y., Zhang X., Mao X. (2014). A comprehensive review of the structure elucidation and biological activity of triterpenoids from *Ganoderma* spp.. Molecules.

[35] Zhao W.Y., Yi J., Chang Y.B., Sun C.P., Ma X.C. (2022). Recent studies on terpenoids in *Aspergillus* fungi: Chemical diversity, biosynthesis, and bioactivity. Phytochemistry.

[36] Zhang P., Zhang Z., Zhang L., Wang J., Wu C. (2020). Glycosyltransferase GT1 family: Phylogenetic distribution, substrates coverage, and representative structural features. Comput. Structrucral Biotechnol. J..

[37] Pichersky E., Raguso R.A. (2018). Why do plants produce so many terpenoid compounds?. New Phytol. Found..

[38] Wright G.D. (2005). Bacterial resistance to antibiotics: Enzymatic degradation and modification. Adv. Drug Deliv. Rev..

[39] Khairullina A., Tsardakas Renhuldt N., Wiesenberger G., Bentzer J., Collinge D.B., Adam G., Bulow L. (2022). Identification and functional characterisation of two oat UDP-glucosyltransferases involved in deoxynivalenol detoxification. Toxins.

[40] Della Gala V., Dato L., Wiesenberger G., Jæger D., Adam G., Hansen J., Welner D.H. (2025). Plant-derived UDP-glycosyltransferases for glycosylation-mediated detoxification of deoxynivalenol: Enzyme discovery, characterization, and *in vivo* resistance assessment. Toxins.

[41] Yuan S., Sun Y., Chang W., Zhang J., Sang J., Zhao J., Song M., Qiao Y., Zhang C., Zhu M. (2023). The silkworm (*Bombyx mori*) gut microbiota is involved in metabolic detoxification by glucosylation of plant toxins. Commun. Biol..

[42] Cadamuro R.D., da Silveira Bastos I.M.A., Silva I.T., da Cruz A.C.C., Robl D., Sandjo L.P., Alves S., Lorenzo J.M., Rodriguez-Lazaro D., Treichel H. (2021). Bioactive compounds from mangrove endophytic fungus and their uses for microorganism control. J. Fungi.

[43] Du H.F., Zhang Y.H., Zhang M., Liu Q.A., Zhu H.J., Cao F. (2022). Marine fungal metabolites as a source of drug leads against aquatic pathogens. Appl. Microbiol. Biotechnol..

[44] Deshmukh S.K., Dufosse L., Chhipa H., Saxena S., Mahajan G.B., Gupta M.K. (2022). Fungal endophytes: A potential source of antibacterial compounds. J. Fungi.

[45] Hridoy M., Gorapi M.Z.H., Noor S., Chowdhury N.S., Rahman M.M., Muscari I., Masia F., Adorisio S., Delfino D.V., Mazid M.A. (2022). Putative anticancer compounds from plant-derived endophytic aungi: A review. Molecules.

[46] Srinivasan R., Kannappan A., Shi C., Lin X. (2021). Marine bacterial secondary metabolites: A treasure house for structurally unique and effective antimicrobial compounds. Mar. Drugs.

[47] Manganyi M.C., Ateba C.N. (2020). Untapped potentials of endophytic fungi: A review of novel bioactive compounds with biological applications. Microorganisms.

[48] Jiang M., Wu Z., Guo H., Liu L., Chen S. (2020). A review of terpenes from marine-derived fungi: 2015–2019. Mar. Drugs.

[49] Yu C.-H., Hermosa G.C., Sun A.-C., Wu C.-W.K., Gao M.-T., Wu C., David Wang H.-M. (2024). Monacolin-K loaded MIL-100(Fe) metal–organic framework induces ferroptosis on metastatic triple-negative breast cancer. Chem. Eng. J..

[50] Cheng T.-H., Lin R.-H., Cheng Y.-S., Shih P.-K., Show P.L., Chen H.-Y., Nakmee P.S., Chang J.-J., Huang D.-M., Wang H.-M.D. (2024). A biomimetic micropillar wound dressing with flavone and polyphenol control release in vitro and in vivo. J. Taiwan Inst. Chem. Eng..

[51] Zhang G., Wang H., Zhang Z., Verstrepen K.J., Wang Q., Dai Z. (2022). Metabolic engineering of *Yarrowia lipolytica* for terpenoids production: Advances and perspectives. Crit. Rev. Biotechnol..

[52] Li Z.J., Wang Y.Z., Wang L.R., Shi T.Q., Sun X.M., Huang H. (2021). Advanced strategies for the synthesis of terpenoids in *Yarrowia lipolytica*. J. Agricutural Food Chem..

[53] Lin P.C., Pakrasi H.B. (2019). Engineering cyanobacteria for production of terpenoids. Planta.

[54] Yang Y.L., Zhang S., Ma K., Xu Y., Tao Q., Chen Y., Chen J., Guo S., Ren J., Wang W. (2017). Discovery and characterization of a new family of diterpene cyclases in bacteria and fungi. Angew. Chem. Int. Ed. Engl..

[55] Liu C., Minami A., Ozaki T., Wu J., Kawagishi H., Maruyama J.I., Oikawa H. (2019). Efficient reconstitution of basidiomycota diterpene erinacine gene cluster in ascomycota host *Aspergillus oryzae* based on genomic DNA sequences. J. Am. Chem. Soc..

[56] Knowles S.L., Raja H.A., Isawi I.H., Flores-Bocanegra L., Reggio P.H., Pearce C.J., Burdette J.E., Rokas A., Oberlies N.H. (2020). Wheldone: Characterization of a unique scaffold from the coculture of *Aspergillus fischeri* and *Xylaria flabelliformis*. Org. Lett..

[57] Adnani N., Chevrette M.G., Adibhatla S.N., Zhang F., Yu Q., Braun D.R., Nelson J., Simpkins S.W., McDonald B.R., Myers C.L. (2017). Coculture of marine invertebrate-associated bacteria and interdisciplinary technologies enable biosynthesis and discovery of a new antibiotic, keyicin. ACS Chem. Biol..

[58] Zhao J., Yang J., Xie Y. (2019). Improvement strategies for the oral bioavailability of poorly water-soluble flavonoids: An overview. Int. J. Pharm..

[59] Elshahawi S.I., Shaaban K.A., Kharel M.K., Thorson J.S. (2015). A comprehensive review of glycosylated bacterial natural products. Chem. Soc. Rev..

[60] Rivas F., Parra A., Martinez A., Garcia-Granados A. (2013). Enzymatic glycosylation of terpenoids. Phytochem. Rev..

[61] Singh G., Dhar Y.V., Asif M.H., Misra P. (2018). Exploring the functional significance of sterol glycosyltransferase enzymes. Prog. Lipid Res..

[62] Seo D.H., Yoo S.H., Choi S.J., Kim Y.R., Park C.S. (2020). Versatile biotechnological applications of amylosucrase, a novel glucosyltransferase. Food Sci. Biotechnol..

[63] Chang T.S., Wang T.Y., Chiang C.M., Lin Y.J., Chen H.L., Wu Y.W., Ting H.J., Wu J.Y. (2021). Biotransformation of celastrol to a novel, well-soluble, low-toxic and anti-oxidative celastrol-29-*O*-beta-glucoside by *Bacillus* glycosyltransferases. J. Biosci. Bioeng..

[64] Wu J.-Y., Ding H.-Y., Wang T.-Y., Tsai Y.-L., Ting H.-J., Chang T.-S. (2021). Improving aqueous solubility of natural antioxidant mangiferin through glycosylation by maltogenic amylase from *Parageobacillus galactosidasius* DSM 18751. Antioxidants.

[65] Chang T.S., Wang T.Y., Yang S.Y., Kao Y.H., Wu J.Y., Chiang C.M. (2019). Potential industrial production of a well-soluble, alkaline-stable, and anti-inflammatory isoflavone glucoside from 8-hydroxydaidzein glucosylated by recombinant amylosucrase of *Deinococcus geothermalis*. Molecules.

[66] Chang T.S., Wu J.Y., Ding H.Y., Tayo L.L., Suratos K.S., Tsai P.W., Wang T.Y., Fong Y.N., Ting H.J. (2024). Predictive production of a new highly soluble glucoside, corylin-7-*O*-beta-glucoside with potent anti-inflammatory and anti-melanoma activities. Appl. Biochem. Biotechnol..

[67] Chang T.S., Wang T.Y., Hsueh T.Y., Lee Y.W., Chuang H.M., Cai W.X., Wu J.Y., Chiang C.M., Wu Y.W. (2019). A genome-centric approach reveals a novel glycosyltransferase from the GA A07 strain of *Bacillus thuringiensis* responsible for catalyzing 15-*O*-glycosylation of ganoderic acid A. Int. J. Mol. Sci..

[68] Chang T.S., Wu J.Y., Wang T.Y., Wu K.Y., Chiang C.M. (2018). Uridine diphosphate-dependent glycosyltransferases from *Bacillus subtilis* ATCC 6633 catalyze the 15-*O*-glycosylation of ganoderic acid A. Int. J. Mol. Sci..

[69] Chang T.S., Chiang C.M., Wang T.Y., Lee C.H., Lee Y.W., Wu J.Y. (2018). New triterpenoid from novel triterpenoid 15-*O*-glycosylation on ganoderic acid A by intestinal bacteria of zebrafish. Molecules.

[70] Chang T.S., Chiang C.M., Kao Y.H., Wu J.Y., Wu Y.W., Wang T.Y. (2019). A new triterpenoid glucoside from a novel acidic glycosylation of ganoderic acid A via recombinant glycosyltransferase of *Bacillus subtilis*. Molecules.

[71] Wu J.Y., Ding H.Y., Wang T.Y., Zhang Y.R., Chang T.S. (2021). Glycosylation of ganoderic acid G by *Bacillus* glycosyltransferases. Int. J. Mol. Sci..

[72] Chang T.-S., Chiang C.-M., Wu J.-Y., Tsai Y.-L., Ting H.-J. (2021). Production of a new triterpenoid disaccharide saponin from sequential glycosylation of ganoderic acid A by 2 *Bacillus* glycosyltransferases. Biosci. Biotechnol. Biochem..

[73] Chang T.-S., Chiang C.-M., Wang T.-Y., Tsai Y.-L., Wu Y.-W., Ting H.-J., Wu J.-Y. (2021). One-pot bi-enzymatic cascade synthesis of novel *Ganoderma* triterpenoid saponins. Catalysts.

[74] Wu J.Y., Ding H.Y., Luo S.Y., Wang T.Y., Tsai Y.L., Chang T.S. (2022). Novel glycosylation by amylosucrase to produce glycoside anomers. Biology.

[75] Lombard V., Golaconda Ramulu H., Drula E., Coutinho P.M., Henrissat B. (2014). The carbohydrate-active enzymes database (CAZy) in 2013. Nucleic Acids Res..

[76] Tian Y., Xu W., Guang C., Zhang W., Mu W. (2023). Glycosylation of flavonoids by sucrose- and starch-utilizing glycoside hydrolases: A practical approach to enhance glycodiversification. Crit. Rev. Food Scienes Nutr..

[77] Zhou W.B., Feng B., Huang H.Z., Qin Y.J., Wang Y.Z., Kang L.P., Zhao Y., Wang X.N., Cai Y., Tan D.W. (2010). Enzymatic synthesis of alpha-glucosyl-timosaponin BII catalyzed by the extremely thermophilic enzyme: Toruzyme 3.0L. Carbohydr. Res..

[78] Moon S.S., Lee H.J., Mathiyalagan R., Kim Y.J., Yang D.U., Lee D.Y., Min J.W., Jimenez Z., Yang D.C. (2018). Synthesis of a novel alpha-glucosyl ginsenoside F1 by cyclodextrin glucanotransferase and its *in vitro* cosmetic applications. Biomolecules.

[79] Gonzalez-Alfonso J.L., Rodrigo-Frutos D., Belmonte-Reche E., Penalver P., Poveda A., Jimenez-Barbero J., Ballesteros A.O., Hirose Y., Polaina J., Morales J.C. (2018). Enzymatic synthesis of a novel pterostilbene alpha-glucoside by the combination of cyclodextrin glucanotransferase and amyloglucosidase. Molecules.

[80] Chang T.S., Wu J.Y., Ding H.Y., Lin H.Y., Wang T.Y. (2024). Exploring gingerol glucosides with enhanced anti-inflammatory activity through a newly identified alpha-glucosidase (*Ar*G) from *Agrobacterium radiobacter* DSM 30147. J. Biosci. Bioeng..

[81] Wu J.Y., Wang T.Y., Ding H.Y., Zhang Y.R., Lin S.Y., Chang T.S. (2021). Enzymatic synthesis of novel vitexin glucosides. Molecules.

[82] Karlova R., Busscher J., Schempp F.M., Buchhaupt M., van Dijk A.D.J., Beekwilder J. (2022). Detoxification of monoterpenes by a family of plant glycosyltransferases. Phytochemistry.

[83] Poppenberger B., Berthiller F., Lucyshyn D., Sieberer T., Schuhmacher R., Krska R., Kuchler K., Glossl J., Luschnig C., Adam G. (2003). Detoxification of the fusarium mycotoxin deoxynivalenol by a UDP-glucosyltransferase from *Arabidopsis thaliana*. J. Biol. Chem..

[84] Lim H.Y., Ong P.S., Wang L., Goel A., Ding L., Li-Ann Wong A., Ho P.C., Sethi G., Xiang X., Goh B.C. (2021). Celastrol in cancer therapy: Recent developments, challenges and prospects. Cancer Lett..

[85] Li M., Xie F., Wang L., Zhu G., Qi L.W., Jiang S. (2022). Celastrol: An update on its hepatoprotective properties and the linked molecular mechanisms. Front. Pharmacol..

[86] Chiang C.-M., Wang T.-Y., Yang S.-Y., Wu J.-Y., Chang T.-S. (2018). Production of new isoflavone glucosides from glycosylation of 8-hydroxydaidzein by glycosyltransferase from *Bacillus subtilis* ATCC 6633. Catalysts.

[87] Chang T.S., Ding H.Y., Wu J.Y., Lin H.Y., Wang T.Y. (2024). Glycosylation of 6-gingerol and unusual spontaneous deglucosylation of two novel intermediates to form 6-shogaol-4′-*O*-*β*-glucoside by bacterial glycosyltransferase. Appl. Enviromental Microbiol..

[88] Chiang C.-M., Wang T.-Y., Wu J.-Y., Zhang Y.-R., Lin S.-Y., Chang T.-S. (2021). Production of new isoflavone diglucosides from glycosylation of 8-hydroxydaidzein by *Deinococcus geothermalis* amylosucrase. Fermentation.

[89] Ding H.-Y., Wang T.-Y., Wu J.-Y., Tsai Y.-L., Chang T.-S. (2022). Enzymatic synthesis of novel and highly soluble puerarin glucoside by *Deinococcus geothermalis* amylosucrase. Molecules.

